# Support Provided by Stop-Smoking Practitioners to Co-users of Tobacco and Cannabis: A Qualitative Study

**DOI:** 10.1093/ntr/ntad115

**Published:** 2023-07-10

**Authors:** Dayyanah Sumodhee, Hannah Walsh, Leonie Brose, Ann McNeill, Andy McEwen, Maria J Duaso

**Affiliations:** Florence Nightingale Faculty of Nursing, Midwifery and Palliative Care (FNMPC), King’s College London, London, UK; Florence Nightingale Faculty of Nursing, Midwifery and Palliative Care (FNMPC), King’s College London, London, UK; Institute of Psychiatry, Psychology and Neuroscience, King’s College London, London, UK; Institute of Psychiatry, Psychology and Neuroscience, King’s College London, London, UK; National Centre for Smoking Cessation and Training, Dorchester, UK; Florence Nightingale Faculty of Nursing, Midwifery and Palliative Care (FNMPC), King’s College London, London, UK

## Abstract

**Introduction:**

Co-use of tobacco and cannabis is highly prevalent among cannabis users and is associated with poorer tobacco cessation outcomes. This study explored the barriers and enablers influencing stop-smoking practitioners’ ability to provide optimal support to co-users.

**Aims and Methods:**

Online semi-structured interviews were audio recorded. Interviewees (*n* = 20) were UK-based certified stop-smoking practitioners. An interview schedule informed by the “capability”, “opportunity”, “motivation” (COM-B) model was designed to explore participants’ perceived barriers and enablers in better supporting co-users to achieve abstinence of both substances or tobacco harm reduction. The transcripts were analyzed using framework analysis.

**Results:**

Capability: Practitioners’ lack of knowledge and skills undermines their delivery of smoking cessation interventions to co-users. Interestingly, when cannabis is used for medicinal reasons, practitioners feel unable to provide adequate support. Opportunity: Service recording systems play an important role in screening for co-use and supporting co-users. When responding to clients’ specific needs and practitioners’ uncertainties, a positive therapeutic relationship and a support network of peers and other healthcare professionals are needed. Motivation: supporting co-users is generally perceived as part of practitioners’ roles but there are concerns that co-users are less likely to successfully stop smoking.

**Conclusions:**

Practitioners are willing to support co-users, but their lack of knowledge and access to an appropriate recording system are barriers to doing so. Having a supportive team and a positive therapeutic relationship is perceived as important. Identified barriers can be mostly addressed with further training to improve tobacco cessation outcomes for co-users.

**Implications**

Supporting cannabis-related abstinence or harm reduction among co-users constitutes an essential part of stop-smoking practitioners’ role. In order to offer adequate support, practitioners need appropriate recording, referral systems, as well as comprehensive training. By prioritizing these measures, practitioners should be able to better assist co-users and improve tobacco cessation outcome.

## Introduction

Worldwide, tobacco and cannabis are two of the most commonly used psychoactive substances that are frequently co-used but rarely co-treated in clinical interventions.^1^ Each substance poses distinct harms, as well as potential aggregated harms.^2^ Despite an overall decline in tobacco consumption in recent decades,^3^ cannabis prevalence appears stable in most of Europe and Australasia, although there is indication that it may be increasing in the United Kingdom and in United States.^4–6^ In Europe, co-administration of tobacco and cannabis is the most common method of cannabis consumption.

The harms of cannabis are exacerbated by its relationship with tobacco, which increase the risk of poorer health-related, psychiatric and psychosocial outcomes.^7^ The use of cannabis is associated with an increased risk of nicotine dependence^8–11^; and international research suggests that it leads to poorer tobacco cessation outcomes.^12–14^ This raises questions about how interventions that are developed for tobacco alone, are being delivered to co-users of tobacco and cannabis. Co-users who do not wish to stop cannabis use can be advised on using different routes of administration for cannabis that do not involve combustion (edibles, concentrates, and vaporizers), which create opportunities for harm reduction.^15^

The type of support provided to smokers wishing to stop-smoking tobacco varies widely between countries.^16^ For instance, Australia and New Zealand provide assistance mainly through free quit lines whilst the UK provides face-to-face support in clinics. Within the United Kingdom, these clinics are organized and managed slightly differently in each country (England, Wales, Scotland, Northern Ireland) but they all provide behavioral support and pharmacotherapy to smokers desiring assistance to quit.^17,18^ This support is typically provided by specialist stop-smoking practitioners who provide tobacco cessation assistance as their only or main role, and community practitioners who provide this type of support secondary to their main role (e.g., community pharmacists, practice nurses, general practitioners [GPs]).

A briefing was published by the National Centre for Smoking Cessation and Training (NCSCT) guiding practitioners on how to support their clients to stop or manage their cannabis use and minimize the impact it has upon tobacco quit attempts.^19^ Nevertheless, our recent study surveying stop-smoking practitioners in the United Kingdom found that practitioners acknowledged the importance of providing smoking cessation support to co-users, however, less than half asked about cannabis use, advised on abstinence of both substances or tobacco harm reduction, or referred cannabis users for support or treatment.^20^

One systematic review reported that the most commonly perceived barriers by staff providing smoking cessation support in drug abuse treatment settings, was their lack of knowledge and training.^21^ However, no studies have conducted an in-depth exploration of the barriers and enablers when delivering smoking cessation support to co-users of tobacco and cannabis. A qualitative study investigated patients’ perceived barriers and enablers on tobacco harm reduction interventions among multiple substance misuse service users but did not include practitioners’ views nor specifically studied tobacco and cannabis co-users.^22^

**Figure 1.**
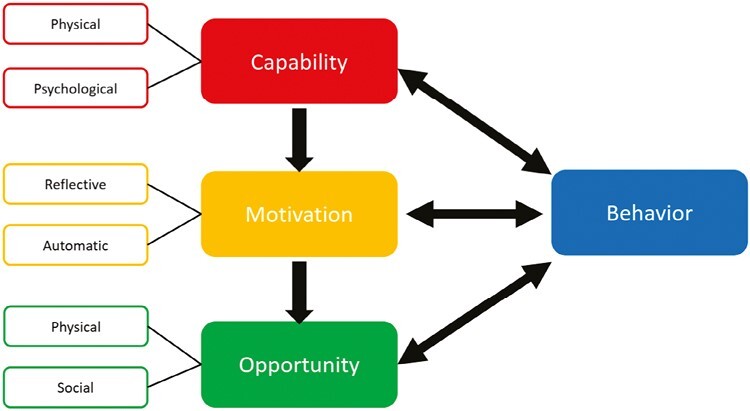
COM-B model (Michie et al.).^23^

The COM-B model^23^ is a theory of behavior that proposes three main components that influence behavior: Capability (physical and psychological), Motivation (reflective and automatic), and Opportunity (physical and social; Figure 1). In other words, for a particular behavior to occur, an individual must have the capability and the opportunity to engage in that behavior and be motivated to enact that specific behavior. ^23^ The model is used to frame barriers and enablers to behavior change thematically and to understand the inter-relationships among them. Previous studies have found that the COM-B model provides a comprehensive framework for describing barriers and enablers of providing smoking cessation support using a qualitative approach.^24–26^

This qualitative study aimed to identify barriers and enablers for stop-smoking practitioners, which included both specialists and community practitioners, when intervening with co-users to improve the smoking cessation outcomes and to structure these barriers and enablers using the COM-B model.

## Materials and Methods

### Design

This study used online semi-structured individual interviews with stop-smoking practitioners and followed COREQ reporting guidelines.^27^ Ethical approval was received from the King’s College Research Ethics Committee (*LRS/DP-20/21-21859*).

#### Patient and Public Involvement (PPI)

Three service users who were co-users of tobacco and cannabis and two stop-smoking practitioners formed a PPI advisory group. The group was consulted regarding the aims and design of the study, helped refine the interview schedule, and provided feedback on the interpretation of the findings and the final themes.

#### Recruitment

As part of our quantitative survey (*n* = 693) on stop-smoking practitioners’ clinical interventions with co-users, conducted in June 2021,^20^ only participants who took part in the survey were invited to take part in a semi-structured interview on the same topic. A total of 291 participants who expressed interest were stratified by self-identified gender (male, female, other, prefer not to say), age groups (18–25, 26–34, 35–44, 45–54, 55–64, 65+) and job roles (specialist stop-smoking practitioner, community practitioner, stop-smoking service manager, stop-smoking service commissioner, other). Within these categories, 20 participants were randomly selected.

The sample size calculation was guided by information power principles defined by Malterud et al.,^28^ which estimates the sample size of a qualitative study based on how much relevant information participants can provide. The recommended sample size for a qualitative study with sufficient information power depends on (1) the aim of the study, (2) sample specificity, (3) use of established theory, (4) quality of dialogue, and (5) analysis strategy. Based on (1) the aim of the current study being broad, (2) sample specificity being moderate, (3) use of the COM-B theoretical framework, (4) moderate quality of dialogue, (e) and a case and cross-case analysis strategy, a sample size of 20 stop-smoking practitioners was considered sufficient.

Potential participants were contacted by the researcher (DS) by e-mail and sent the information sheet about the qualitative study. All participants approached agreed to participate in the study, signed a consent form prior to the interview and were offered a £20 shopping voucher as compensation with the option to donate it to Cancer Research UK.

#### Interviews

The COM-B model was used to guide the interview schedule to explore stop-smoking practitioners’ beliefs regarding supporting co-users of tobacco and cannabis. Table 1 presents examples of the interview questions, and Supplementary Appendix 1 contains the full list of questions. The interview was piloted with the first participant, after which the order of the questions was slightly changed to improve the flow of the interview. Interviews lasted 45 to 60 minutes and were conducted online between July and September 2021 by DS. All interviews were audio-recorded and transcribed in verbatim.

**Table 1.** Prompt for Interviews According to the COM-B Model

COM-B	Examples of interview questions and prompts
Capability	
Psychological/Physical capability	What are your thoughts on your level of knowledge and skills when advising clients who smoke tobacco and also use cannabis?
Opportunity	
Physical opportunity	Does your service provide its own treatment guidance for cannabis use?
Social opportunity	How supportive is your team and colleague if you have questions or uncertainties about supporting co-users of tobacco and cannabis?
Motivation	
Reflective motivation	How important is it for you to support co-users of tobacco and cannabis to stop smoking?
Automatic motivation	How often do you ask clients about their current cannabis use?

#### Analysis

Transcripts were analyzed using framework analysis, applying the COM-B model. This method starts deductively with a priori codes from the study’s aims and objectives and the analysis is subsequently inductive.^29^ The researcher (DS) read and reread the transcripts for familiarization and data immersion. Three transcripts were then independently coded by DS, HW, and MD to create an initial coding and thematic framework based on the COM-B model to identify themes relating to the barriers and enablers to of engagement with co-users.

Comparisons were made between transcripts based on the thematic framework. Discrepancies throughout the analytical process were related to the assignments of codes to specific COM-B components, namely the Physical Capability or Social Opportunity themes and these were resolved by consensus with the co-authors. All transcripts were then coded line-by-line by DS using NVivo 1.6.1. The themes identified were subsequently mapped (by DS) onto the COM-B model constructs, where applicable, to generate analytical themes with the coding framework undergoing minor refinements as required during this process. The mapping was discussed and agreed with DS, MD, and HW to assure codes that could be interpreted as being more than one function of COM-B were allocated to the most appropriate COM-B domain. The themes were then presented to the PPI advisory group who provided feedback on the interpretation of the findings and the final themes.

#### Reflexivity

All authors had good knowledge of the COM-B model. MD, HW, AM, LB, and AMc are researchers in tobacco addiction but do not provide smoking cessation support as part of their role. DS worked as a stop-smoking practitioner for 8 years. Thus, being mindful of potential bias as a researcher, she kept notes and regularly checked with the team to discuss bias and the codes and themes being identified. Furthermore, the co-authors aimed to ensure that both barriers and enablers associated with advice provision were discussed in a balanced manner and their interpretations of the findings were in line with the views of the practitioners in the PPI advisory group.

## Results

The 20 participants comprised 15 practitioners with experience in advising co-users of tobacco and cannabis and 5 with no experience advising co-users (Table 2). Fifteen participants were female and five were male, representing a similar gender distribution of practitioners in the UK in previous studies.^30–32^ Most worked as community practitioners (75%) and 16 worked in England, 2 in Wales, and 2 in Scotland.

**Table 2.** Participant’s Characteristics (*n* = 20)

Participants’ characteristics	*n* (%)
Gender	
Female	15 (75)
Male	5 (25)
Age	
18-25	1 (5)
26-34	6 (30)
35-44	4 (20)
45-54	5 (25)
55-64	4 (20)
Role	
Specialist practitioners	7 (35)
Midwife	3 (15)
Pharmacist	2 (10)
Mental health nurse	2 (10)
General practitioner (GP)	2 (10)
Wellbeing practitioner	2 (10)
Stop-smoking service manager	1 (5)
Dentist	1 (5)
Experienced advising co-users	
Yes	15 (75)
No	5 (25)
	Mean (SD)
Years of experience	6.8 (6.0)

Table 3 outlines the final analysis with the deductively coded themes of the COM-B model and the subsequently inductively coded sub-themes to explore the barriers and enablers of supporting co-users of tobacco and cannabis. These findings are described in greater detail below. No themes were identified in the “Physical Capability” component. The term “co-user” refers, in this paper, to users of both tobacco and cannabis.

**Table 3.** Deductively Coded Themes Mapped Onto the COM-B Model and Inductively Coded Sub-themes Exploring the Barriers and Enablers of Supporting Co-users of Tobacco and Cannabis

Deductively coded themes mapped on to COM-B model	Inductively coded sub-themes
Capability^1^	
1. Psychological Capability Participants’ mental functioning (eg, understanding) involved when advising	1.1. Knowledge and understanding of interventions for co-users
	1.2. Opening up multiple complex issues
	1.3. Experience with co-users fosters confidence
Opportunity	
2. Physical Opportunity Opportunity for practitioners to advise that involves inanimate parts of the environmental system and time	2.1. Having a recording system in place
	2.2. Time constraints
	2.3. Use of stop-smoking medication with co-users
3. Social Opportunity Opportunity for practitioners to advise that involves another person/organization	3.1. Collaborating with peers, multi-disciplinary teams and addiction specialists
	3.2. Therapeutic relationship with co-users
	3.3. Building a connection remotely
Motivation	
4. Reflective Motivation Practitioners’ motivation to advise that involves conscious thought processes	4.1. Role identity
	4.2. More challenging for co-users to successfully achieve tobacco cessation
5. Automatic Motivation Practitioners’ motivation to advise that involves habitual and instinctive processes	5.1. Tobacco cessation intervention protocol is adaptable for co-users

^1^No themes were identified under the “Physical Capabilities” component.

### Capability

#### Psychological Capability

##### Knowledge and Understanding of Interventions for Co-users.

Practitioners expressed their willingness to address abstinence or harm reduction among co-users, but expressed concern about their level of knowledge on the topic: “*I would like to have more training in it, because the knowledge I have, I don’t know whether that’s going to be fully correct”* (P1, 4 years of experience, pharmacist).

When asked what future training would be helpful, suggestions included e-learning modules covering: “*How tobacco and cannabis interact with each other, the cost, the effects it has on a user and motivators to start using it*” (P7, 3 years, mental health nurse). Information regarding the potential risks associated with cannabis consumption such as how it impaired users’ concentration was also requested. Practitioners expressed the need to learn how best to explain to clients the purpose of enquiring about and recording their cannabis use, and to inform them about confidentiality.

Dedicated clinical guidance for practitioners was recommended to guide practitioners on the treatment plan for co-users including optimal length of treatment, appropriate behavioral and pharmacological support (if any). Overall, whilst most practitioners were motivated to support co-users, some preferred referring clients to resources due to their lack of knowledge:

“*I would like to be able to give them a leaflet because I wouldn’t feel comfortable talking about it…I can tell you whatever you want to know about the patches and gums and lozenges and Champix…but it’s a little bit more of a mystery to me when it’s comes to cannabis*” (P13, 2 years, GP)

##### Opening Up Multiple Complex Issues.

Practitioners perceived that cannabis use was often associated with a wider set of problems and this resonated with our PPI advisory group. For instance, although consumption of cannabis can be a recreational practice, it could also be used to manage pain, sleep, or psychosocial issues. Some practitioners described the need to assess and address these issues before stopping smoking, which felt beyond the remit of their role:

*“One woman had arthritis, she used it for pain relief, so then that’s just educating them around how they can get the same effect from using other substances rather than cannabis but I’m not qualified for that” (*P2, 3 years, specialist practitioner)

##### Experience With Co-users Fosters Confidence.

Our sample included 15 practitioners who had experience in advising co-users of tobacco and cannabis and 5 had no experience advising co-users. The 15 participants with more frequent interactions with co-users in their current or previous work, for example in drug and addiction services, tended to feel more comfortable talking about cannabis use and in building trust with the co-users. In contrast, the five practitioners with fewer encounters with co-users generally felt less confident leading to less positive therapeutic relationships. These practitioners, instead, would refer their co-user clients to more experienced colleagues:

*“I mean it’s not like every single patient that comes in for smoking cessation has got a cannabis problem. We have, three technicians who are really good, I might ask them for advice or even just refer the patient to them”* (P4, 2 years, pharmacist).

### Opportunity

#### Physical Opportunity

##### Having a Recording System in Place.

All practitioners were willing to use a system to record cannabis use. Some practitioners noted they did not systematically ask about cannabis use since it was not included in the mandatory assessment. Hence having a recording process where clients’ cannabis use was a mandatory question would prompt them to ask about it and ensure they recorded all key information should the client need to be seen by a different practitioner. They also reflected that co-users might be reluctant to talk:

*“I don’t think the questions are mandatory so I wouldn’t ask about cannabis use unless they brought it up…when it comes to illicit drugs, people are not so forthcoming because they think you’re going to get them into trouble or tell on them.”* (P3, 8 years, specialist practitioner).

Some practitioners also suggested that current recording systems could be better structured:

“*It would make it easier if there was more structured way to ask questions, so there’s a tick box for “Do they smoke cannabis? and if you tick “Yes” then there’s a dropdown list “OK, these are the things you might want to discuss with them*” (P1, 4 years, pharmacist)

##### Time Constraints.

Time constraints hindered practitioners’ work with co-users as they “*do generally need a little bit more support”* than tobacco users only (P18, 8 years, specialist practitioner). Practitioners believed that stopping tobacco for co-users could be more difficult due to the increased levels of tobacco dependence. Furthermore, treatment needed to address behavior changes for cannabis use as well as for tobacco use.

To mitigate this, practitioners recommended longer appointments slots or the introduction of a referral system to drug specialist teams: “*You’re cramming in as much information as you can so you think, do I have time to go into it now or could I, like sort of safety net it and refer it”* (P5, 11 years, specialist practitioner).

##### Use of Stop-Smoking Medication with Co-users.

Although there is no pharmacological treatment available for cannabis cessation, nicotine replacement therapy (NRT) products were still seen as effective for the behavioral aspect:

*“We do recommend NRT, perhaps it might help; not with the cannabis, but at least with the hand-to-mouth habits, something like an inhalator”* (P14, 8 years, GP).

Some practitioners noted that using varenicline with co-users “*has an impact on their cannabis use, they said at the beginning they don’t want to reduce their cannabis use but they find that with Champix it just seems to happen anyway*” (P15, 4 years, specialist practitioner).

#### Social Opportunity

##### Collaborating With Peers, Multi-Disciplinary Teams, and Addiction Specialists.

Practitioners stressed the importance of providing an efficient and comprehensive support to co-users by collaborating with peers and liaise with multiple services.

For less confident practitioners, more experienced colleagues were their first point of contact if they have questions or safeguarding concerns: “*If there was a problem with a client I could say: ‘Look I don’t have the information, but I can speak to my manager’*” (P16, 6 years, specialist practitioner).

Furthermore, since clients were often unable to receive detailed information on cannabis cessation from stop-smoking practitioners, being able to talk anonymously to their GPs so they can be referred for specialist support was essential:

“*So a GP knowing about their smoking is one thing but knowing about their drug use, it could affect their career in some way. People are actually happy to talk as long as it’s confidential”* (P7, 3 years, mental health nurse)

This also emphasized the need for practitioners to be aware of the referral system pathway for specialized support for cannabis cessation. However, few practitioners knew how to refer their clients and provided leaflets about how to self-refer.

##### Therapeutic Relationship With Co-users.

A positive therapeutic relationship was paramount to making co-users feel comfortable with talking and receiving support. Practitioners were aware of the need to build a level of trust with their clients in order to talk about cannabis use: “*You can’t be judgemental, because if you are, you’re going to push all the patients away. They’re going to not want to speak to you about it [cannabis].”* (P8, 6 years, nurse).

##### Building a Connection Remotely.

Since the Covid-19 pandemic, practitioners had been mostly providing stop-smoking support by telephone rather than face-to-face. This could have impaired the opportunity to build a meaningful connection with clients, which was seen as particularly important when treating issues such as illegal drug use: *“Having the patient right in front of you makes it easier to see face expressions or the body language, you can talk more openly…especially something sensitive like cannabis use.”* (P5, 11 years, specialist practitioner)

### Motivation

#### Reflective Motivation

##### Role Legitimacy.

For one stop-smoking service manager, although the primary focus was tobacco cessation, but they also embraced and supported approaches for tobacco harm reduction and abstinence of both substances as it could impact on their clients’ quit attempts:

“*You need to look across all a person’s risky behaviours and it directly impacts on their quit attempt. Although we’re based on the number of quits in terms of smoking [tobacco] – they [tobacco and cannabis] are inter-related, we can’t really separate the two*.” (P9, 15 years, stop-smoking service manager)

Practitioners agreed that addressing abstinence of both substances or tobacco harm reduction was part of their role. However, a number felt they were not encouraged to pursue working with co-users as it seems that their role identity differed from the service’s referral policy: “*I keep getting reminded by my manager that we’re not a stop-cannabis smoking agency. So, I’m referring on to the local drug service within the area that they live.”* (P2, 3 years, specialist practitioner).

##### Cannabis Use Impact on Tobacco Cessation Outcome.

Many practitioners believed that stopping smoking was more challenging for co-users of tobacco and cannabis than for tobacco-only users. They consequently thought that co-users had a lower likelihood of successfully quitting:

“*I think it makes things more difficult. So if somebody is smoking both or using both substances, they will often falter or switch between, rather than stopping long term*” (P10, 3 years, specialist practitioner).

#### Automatic Motivation

##### Tobacco Cessation Intervention Protocol is Adaptable for Co-users.

The fact that practitioners can adapt the standard smoking cessation intervention for co-users gave them a sense of re-assurance that they could follow a treatment protocol with which they were familiar:

*“I’d try to take the same approach as I would with somebody trying to give up smoking so if we focus on the stop-smoking side of things, then it’s very similar then that’s fine, I’ll give them some NRTs or Champix”* (P4, 2 years, pharmacist).

However, some practitioners expressed uncertainties around the protocol to follow should their clients wish to stop one substance: *“If someone wanted to quit both, we can just tailor our goals to smoking in general, whereas if you’re just going to quit one of it, then how do you plan the sessions?”* (P1, 4 years, pharmacist).

## Discussion

This study provides new insight into the barriers and enablers for delivering stop-smoking support to co-users to achieve abstinence of both substances or tobacco harm reduction. There has been a previous lack of focus on the perspectives of stop-smoking practitioners on the support they provide to co-users. Framing their experiences in all three components of the COM-B model demonstrates the areas where interventions can be targeted to improve the support that practitioners provide to co-users.

Enablers for practitioners’ capability were centered around their confidence in advising co-users. Frequently seeing co-users in their service or having previous experience working with them allowed practitioners to feel more comfortable and knowledgeable on how to support them. By extension, this meant that the more experienced practitioners were, the better they were able to build positive therapeutic relationships with their clients. Our findings are consistent with van de Ven et al. who demonstrated that clinicians’ years of experience was associated with better relationship with patients when delivering drugs and alcohol interventions.^33^ The barriers identified were practitioners’ lack of knowledge on how to deliver intervention to co-users. Here, participating in evidence-based training courses and having access to resources could as suggested by previous research improve smoking cessation rates from the NCSCT.^34^ Our study suggested that clients might be consuming cannabis for reasons that practitioners are not trained to treat, such as sleep or pain issues. The health issues of medicinal cannabis users’ need to be addressed with qualified healthcare professionals to increase their chances of successfully quitting.

In terms of opportunity, having an appropriate recording system in place which would remind practitioners to ask about cannabis use and flag appropriate questions for co-users was identified as an important enabler. The adoption of the International Cannabis Toolkit for example, which consists of a three-layered hierarchical pyramid (universal, detailed self-report and biological measures) would facilitate consistent information for all clients across the different settings.^35^ Involving peers or other healthcare professionals was an opportunity to maximize the support. Previous findings suggest that reaching out to other healthcare professionals enabled co-users to receive more comprehensive support to address their cannabis use and this follows a similar multidisciplinary approach to alcohol addiction treatment in acute hospitals.^36,37^ However, in our study practitioners reported that referring to other healthcare professionals was complex due the absence of a referral or self-referral system and concerns over confidentiality. Time constraints were considered a barrier due to the lack of capacity to offer longer appointments. A positive therapeutic relationship and a non-judgmental approach were fundamental in treating co-users. This corroborates findings by Richter et al. indicating that a positive therapeutic relationship and a non-judgmental approach play a key role in smoking cessation interventions for people in substance misuse treatment.^22^ Although there is no pharmacological support for cannabis cessation, providing tobacco cessation medications could alleviate cannabis withdrawal symptoms.

Most practitioners felt motivated to support co-users as they felt this fitted their role. While their primary focus was tobacco cessation, they were willing to support co-users but as identified above, a clear referral pathway was needed for specialized cannabis cessation support. Practitioners were re-assured that they could adapt the treatment protocol for tobacco cessation, with which they were familiar. However, their motivation appeared diminished when they lacked knowledge on how to support co-users. Furthermore, practitioners perceived the delivery of interventions as challenging due to co-users’ higher level of addiction and lower chances of stopping smoking.^12–14^ Practitioners in our study suggested that providing some nicotine replacement products could alleviate their clients’ cannabis withdrawal symptoms. This is in line with new evidence showing that pharmacological treatment for tobacco such as patches^38^ and varenicline^39–41^ may assist with cannabis withdrawal symptoms.

Our study results should be considered in light of a few limitations. Our sampling method could have introduced bias into our findings, as only participants who completed our initial online survey were recruited for the interviews. Hence, it is possible that the participants had a particular interest in or experience of working with co-users. While the COM-B model was a helpful framework for analysis, we faced challenges in categorizing the codes into a singular category (capability, opportunity, motivation) when they involved multiple concepts.

## Implications

Practitioners recognized the potential to address cannabis-related abstinence or harm reduction with co-users as part of their role. This can be achieved and practitioners should be equipped with an appropriate recording system that considers co-users and requires mandatory structured recording of clients’ cannabis use, so that consistent information regarding clients’ needs is available. In addition, a clear referral and self-referral pathway to specialized services or healthcare professionals and where possible, longer appointments slots should be provided. Practitioners confidence could be enhanced with a comprehensive training programme on how to design a treatment plan with co-users and convey information about confidentiality related to clients’ cannabis use. Future training should also consider medicinal cannabis use.

## Conclusion

Stop-smoking practitioners are willing to support co-users to stop smoking but the lack of knowledge and appropriate recording system for cannabis might undermine the support they can provide. Practitioners considered a supportive team and a positive therapeutic relationship with co-users as particularly important for an effective intervention delivery. Future training and health policy measures need to address each of the identified barriers for an improved tobacco cessation outcome among co-users.

## Supplementary Material

ntad115_suppl_Supplementary_Material

## Data Availability

The participants of this study did not give written consent for transcripts of the data to be shared publicly. Excerpts of the transcripts relevant to the study are available within the paper, further excerpts can be made available upon reasonable request to the corresponding author (DS).
