# Supplementary material for: Support Provided by Stop-Smoking Practitioners to Co-users of Tobacco and Cannabis: A Qualitative Study
Source: Nicotine Tob Res. 2023 Jul 10;26(1):23–30. doi: 10.1093/ntr/ntad115 (PMC10734383; doi:10.1093/ntr/ntad115)
Supplement: ntad115_suppl_Supplementary_Material [file ntad115_suppl_supplementary_material.docx]

Qualitative study appendix

APPENDIX 1: INTERVIEW SCHEDULE

- - - 1. Can you tell me about the training (if any) you have received to support tobacco and cannabis users to quit smoking?
      2. What are your thoughts on your level of knowledge and skills when advising clients who smoke tobacco and also use cannabis?
      3. How often do you ask clients about their current cannabis use?
      4. Could you please describe how you ask clients about their current cannabis use?
      5. How comfortable are you about talking to clients about cannabis use?
      6. Do you have experience advising clients who smoke tobacco and also use cannabis?
      7. How important is it for you to support co-users of tobacco and cannabis to stop smoking?
      8. How do you explore client’s perceived effect of cannabis cravings on tobacco cessation and vis versa?

- - - 1. What makes it easier to assess cannabis use and provide support for clients who use both substances?
- What makes it difficult to assess cannabis use and provide support for clients who use both substances?
- What could be done to make it easier?
  - - 1. What would you do if one of your clients who smokes cannabis might be putting others and themselves in danger? (eg. operating heavy machinery)
      2. Does your service provide its own treatment guidance for cannabis use? If yes, would it be possible to share it with us?
      3. How supportive is your team/colleague if you have questions/uncertainties about supporting co-users of tobacco and cannabis?
      4. We have 2 clinical scenarios, and we would like to know what recommendations you would give to the following clients:

Clinical scenario 1: A 27-year-old woman comes in for her first session to get help to stop smoking. She smokes 5 joints of cannabis and tobacco and around 10 cigarettes daily. She is 8-weeks pregnant and her midwife recommended her stopping smoking. She has never tried to stop before and would like to get your support to stop smoking both substances.

Clinical scenario 2: A 32-year-old man smokes up to 10 joints of cannabis and tobacco daily but doesn’t smoke cigarettes. He has COPD and his doctor advised him to stop smoking. He has tried to quit smoking twice before but was unsuccessful. It’s his first session with you and he said he wants to quit smoking tobacco but doesn’t want to stop smoking cannabis.

- - - 1. Is there anything else you would like to mention or emphasize?
